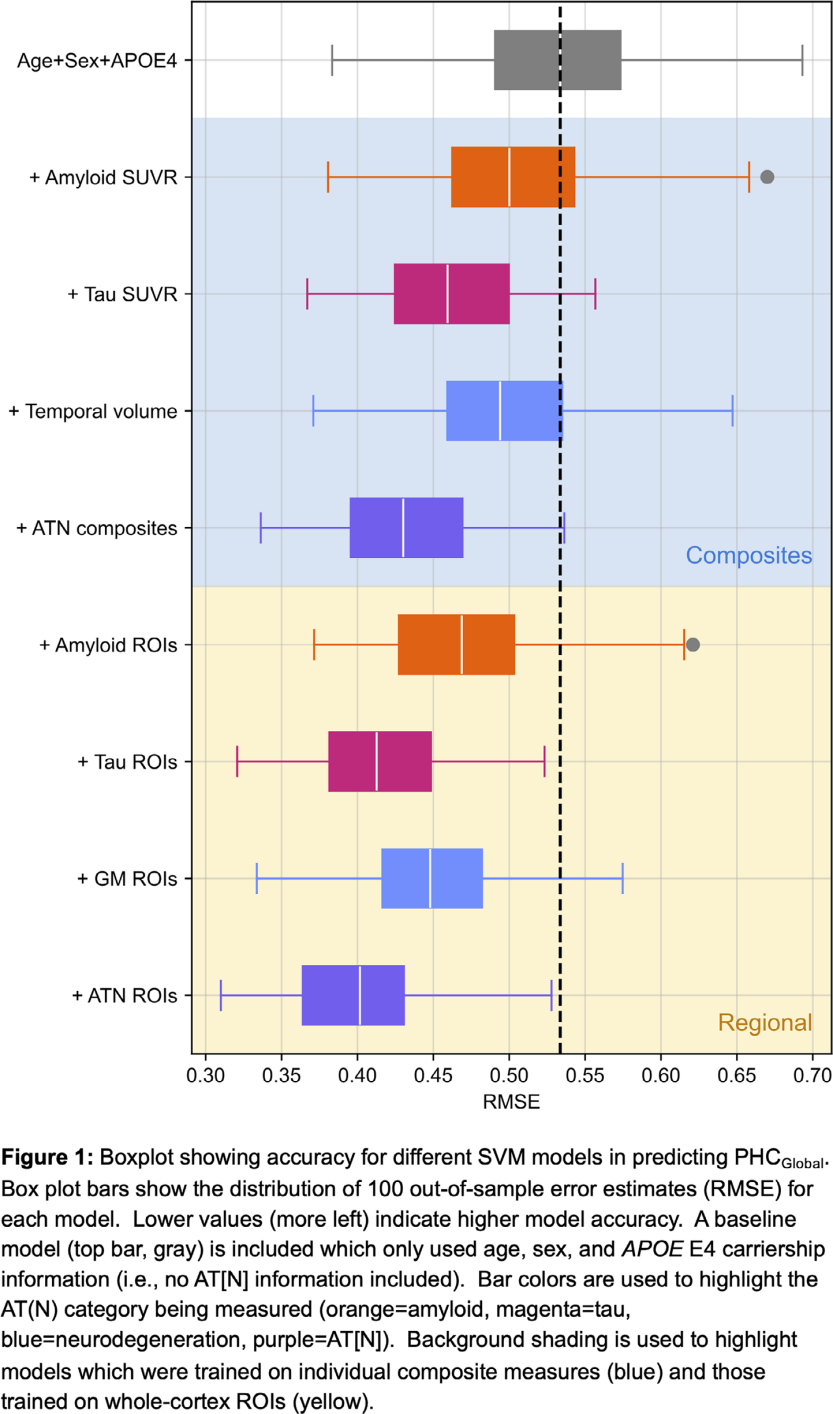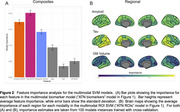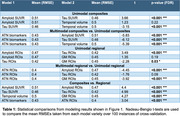# Regional AT(N) information improves cognitive prediction in machine learning models

**DOI:** 10.1002/alz70856_107310

**Published:** 2026-01-08

**Authors:** Tom Earnest, Braden Yang, Aristeidis Sotiras

**Affiliations:** ^1^ Mallinckrodt Institute of Radiology, Washington University School of Medicine in St Louis, St Louis, MO, USA; ^2^ Institute for Informatics, Washington University, St. Louis, MO, USA

## Abstract

**Background:**

Often, imaging studies of Alzheimer's Disease (AD) use composite regions of interest (ROIs) to define global measures of AT(N) (amyloid/tau/neurodegeneration) pathology severity. Such composite ROIs necessarily span a relatively small part of the cortex, potentially omitting pathological signal in other parts of the brain. Here, we evaluated how composite AT(N) measures compare to ROIs spanning the whole brain in prediction of cognitive performance.

**Method:**

We selected 473 individuals from ADNI who underwent cognitive testing, MRI, and PET (florbetapir and flortaucipir). Our target measure of interest was global cognitive performance (PHC_Global_: average of all domain scores from the Phenotype Harmonization Consortium). We selected composite (amyloid: Centiloid ROI SUVR, tau: meta‐temporal SUVR, neurodegeneration: meta‐temporal volume) and regional (SUVRs/volumes in 68 cortical gray matter ROIs) measures of AT(N) pathology. We trained support vector machines (SVMs) to predict PHC_Global_ using either composite or regional features. In each case, we trained models with only one AT(N) category (unimodal) and all three (multimodal). All models also included age, sex, and *APOE* E4 carriership as features. A baseline model only included these demographic features. Models were trained using a repeated, nested cross‐validation scheme with 10 repeats and 10 outer folds. We used Nadeau‐Bengio t‐tests to compare the accuracy (root mean squared error, RMSE) of trained models in out‐of‐sample folds. For multimodal models, we derived feature importance estimates by computing the covariance of each feature with PHC_Global_.

**Result:**

For both unimodal and multimodal models, SVMs trained on regional AT(N) information were consistently more accurate than versions trained on composite measures in predicting cognitive performance (Figure 1, Table 1). For regional models, the unimodal tau SVM and the multimodal SVM were more accurate than unimodal amyloid or neurodegeneration models. Visualizations of feature importance highlighted the relative extra weighting of tau for both biomarker and ROI SVMs (Figure 2).

**Conclusion:**

We found that machine learning models which included ROIs spanning the whole brain were consistently more accurate than models including common global biomarker values when predicting cognitive performance. Our results indicate that imaging features outside of standard composite regions may be useful for assessing the biological progression of AD.